# Chemotherapy May Obviate Prophylactic Femoral Nail Surgery for Multiple Myeloma Patients With High Mirels’ Score Lesions and Impending Pathological Hip Fracture

**DOI:** 10.7759/cureus.37670

**Published:** 2023-04-17

**Authors:** Omkaar Divekar, Bisola Ajayi, Ben Barkham, Jason Bernard, Tim Bishop, Yasmin Reyal, Fenella Willis, Kevin Boyd, Darren Lui, Ross Coomber

**Affiliations:** 1 Trauma and Orthopaedics, St. George's University Hospital, London, GBR; 2 Orthopaedics, St. George's University Hospital, London, GBR; 3 Complex Spinal Surgery, St. George's University Hospital, London, GBR; 4 Hematology and Oncology, St. George's University Hospital, London, GBR; 5 Haematology, Royal Marsden Hospital, London, GBR; 6 Orthopaedics, St. George's Hospital, London, GBR

**Keywords:** mirels' score, pathological fracture, vtd therapy, femoral nail, multiple myeloma treatment

## Abstract

Bone involvement presents in >80% of patients with multiple myeloma. This causes lytic lesions for which prophylactic surgery is indicated to prevent pathological fractures if the lesion is graded ≥9/12 on Mirels’ score. Although successful, these surgeries have risks and extended recovery periods. We present a case indicating myeloma chemotherapy may obviate prophylactic femoral nailing for high Mirels’ score lesions in the femoral head with impending pathological hip fracture.

A 72-year-old woman presented in December 2017 with back pain. A plain X-ray indicated degenerative anterolisthesis in her lumbosacral spine. Serum analysis revealed abnormal protein, globulin, alkaline phosphatase, and albumin levels while protein electrophoresis and serum immunofixation revealed raised immunoglobulin A (IgA) kappa paraprotein and kappa serum free light chains, respectively. Whole-body CT scans showed widespread lytic bone lesions and bone marrow biopsy confirmed infiltration by plasma cells. She was diagnosed with International Staging System (ISS) stage 3 multiple myeloma, which was successfully treated with bortezomib, thalidomide and dexamethasone with regular bisphosphonates that year. She presented again to the hospital in June 2020 with acute back and pelvic pain; Her paraprotein and serum-free light chains had increased significantly from her previous clinic appointment, indicating serological progression. MRI showed a relapse of the myeloma deposits in her right femoral head and spine. The deposit in her femoral head was graded 10/12 on Mirels’ score, which indicated prophylactic femoral nailing. Instead, the patient was treated with daratumumab, bortezomib, and dexamethasone with escalation to monthly zoledronic acid infusions, as it was thought surgery would provide limited cytoreductive effect, preventing chemotherapy for six weeks post-surgery, potentiating pathological hip fracture and disease progression at other sites. This resulted in a complete response, thus reducing the deposits such that the femoral lesion was graded <8 on Mirels’ score, improved her pain, and restored her ability to traverse stairs. She remains in complete response with ongoing daratumumab and denosumab maintenance treatment as of December 2022.

Chemotherapy and bisphosphonates substantially reduced the myeloma deposit in the femoral head such that indications of prophylactic surgery were eliminated according to Mirels’ score recommendations. This reduced the risk of pathological hip fracture whilst eliminating surgical complications. Further research should be conducted into the safety and efficacy of this treatment regimen in patients with high Mirels’ score lesions. With this knowledge, consideration can be taken as to whether prophylactic femoral nailing is necessary given strong indications.

## Introduction

Bone involvement is present in >80% of patients with multiple myeloma [1]. This can cause lytic lesions in the bone leading to pathological fractures and significant pain. Prophylactic orthopaedic surgery e.g. vertebroplasty, kyphoplasty and stabilisation for spinal lesions and nailing for long bone stabilisation [2], is indicated to prevent pathological fractures if the lesion is graded ≥9/12 on Mirels’ score [3]. Although successful, these surgeries have risks and lengthy recovery periods. Recent advances in the non-surgical management of multiple myeloma, including targeted chemotherapy, have been shown to be effective at preventing the osteoclastogenic processes occurring within the microenvironment of the myeloma deposits, thus inhibiting their growth [4]. We present a case indicating that myeloma chemotherapy may obviate prophylactic femoral nailing for high Mirels’ score lesions in the femoral head with impending pathological hip fracture.

This article was previously presented as a poster at the 6th World Congress on Spine and Spinal Disorders on December 6, 2020.

## Case presentation

A 72-year-old woman presented to the hospital in December 2017 with back pain. A plain X-ray of the patient’s lumbar spine indicated an advanced generalised loss of bone density and degenerative anterolisthesis in the lumbosacral region of her spine (L5-S1). Her primary care physician performed routine investigations for her back pain, which found her to be anaemic. Subsequent serum analysis indicated elevated levels of total protein - 81g/L (60 - 80g/L), globulin - 52g/L (20 - 40g/L) and alkaline phosphatase - 199U/L (30 - 130U/L) and decreased albumin levels - 29g/L (35 - 50g/L). Protein electrophoresis and serum immunofixation revealed an immunoglobulin A (IgA) kappa paraprotein of 18g/L (0.8 - 4.0g/L) and raised kappa serum free light chains of 2000mg/L (3.30 - 19.40mg/L). Whole-body CT scans showed widespread lytic bone lesions and a bone marrow biopsy confirmed infiltration by plasma cells with normal FISH (fluorescent in-situ hybridisation). She was hence diagnosed with International Staging System (ISS) stage 3 multiple myeloma [5] in March 2018. This was successfully treated that year with bortezomib, thalidomide and dexamethasone in addition to regular bisphosphonates (zoledronic acid). According to the International Myeloma Working Group (IMWG) criteria, she achieved a stringent complete response to this initial induction therapy [6], which was subsequently discontinued, however, she was deemed unsuitable for stem cell transplant due to recurrent diverticulitis. As such, she was placed on active surveillance.

**Figure 1:**
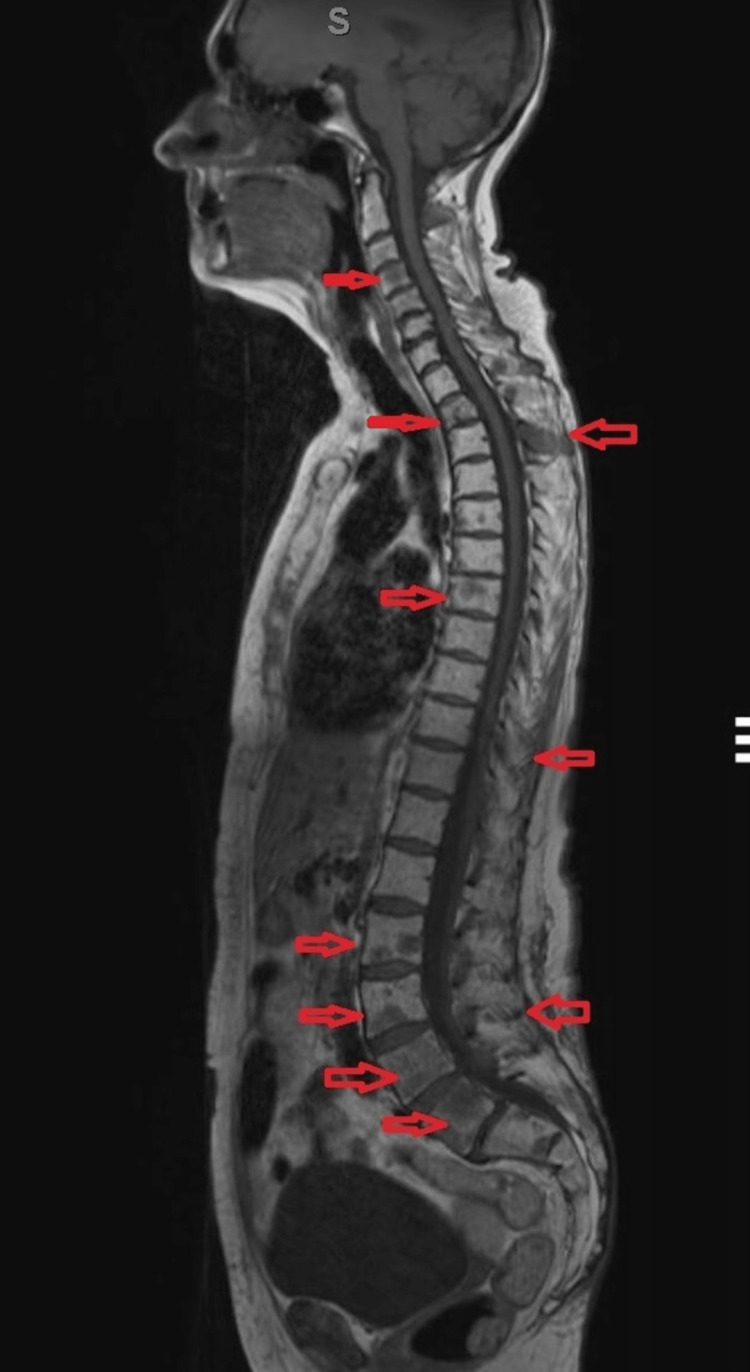
MRI lumbar-sacral spine T1-weighted image showing panspinal multiple myeloma lesions

**Figure 2:**
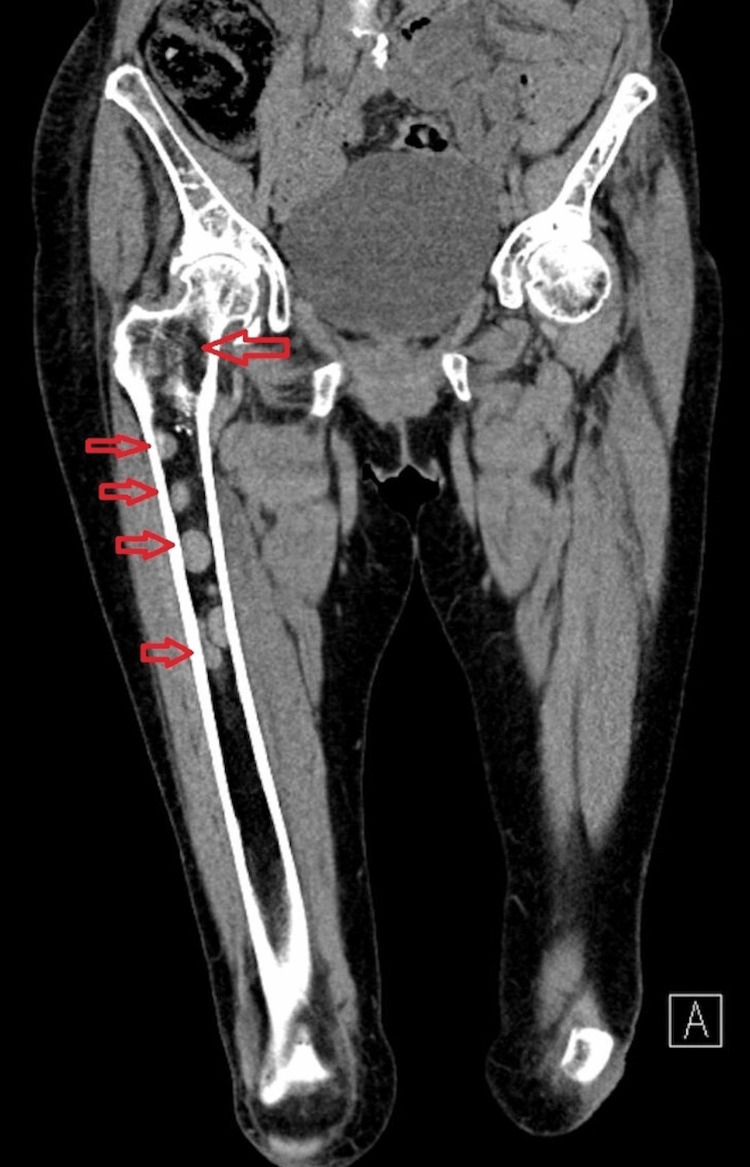
CT scan coronal plane image showing right hip and femur with several multiple myeloma lesions

The patient presented again to the hospital in June 2020 with rapid onset severe bone pain in her back and pelvis. Her paraprotein had increased from 0g/L at her previous clinic appointment to 9g/L while her serum-free light chains had also increased from 14mg/L to 103mg/L, indicating serological progression. Whole-body MRI scans indicated a very aggressive relapse of the myeloma with deposits in her spine and right femur with significant involvement of the bony cortex as seen in Figure 1 and Figure 2. The deposit in her femoral head was graded 10/12 on Mirels’ score indicating prophylactic femoral nailing. However, it was thought that surgery would provide a limited cytoreductive effect and prevent chemotherapy from occurring for six weeks post-surgery, potentiating pathological hip fracture and allowing for disease progression at other bony sites. As early, rapid treatment is vital, the patient was instead treated with daratumumab, bortezomib and dexamethasone with an escalation of zoledronic acid to monthly infusions. The patient was also kept ambulatory with protected weight-bearing, crutches, physiotherapy and fall-prevention education.

These interventions alone resulted in a complete response according to the IMWG uniform criteria [6] as well as the resolution of all focal active bone marrow lesions on whole-body diffusion-weighted MRI scans. Furthermore, the myeloma deposits were significantly reduced such that the femoral lesion was graded <8 on Mirels’ score. Overall, these non-surgical interventions led to a significant reduction in the patient’s night and rest pain in her lumbosacral spine and pelvis. Prior to treatment, the patient was unable to traverse the stairs, however, within three months of beginning treatment, she was able to do so. She remains in a complete response as of December 2022 with ongoing daratumumab and denosumab (120mg) maintenance treatment (once every four weeks) with surveillance blood tests performed concurrently. She is fully ambulatory and reports no symptoms relating to her femoral lesion.

## Discussion

Multiple myeloma makes up 1% of all cancers diagnosed worldwide [7]. Its incidence increases with age and is positively associated with the male sex and black ethnicity [8]. In newly diagnosed myeloma, current treatment algorithms distinguish patients based on their eligibility for autologous stem cell transplant (ASCT) [9]. For those who are eligible, initial treatment involves four to six cycles of induction therapy with a combination of bortezomib (Velcade), dexamethasone and either lenalidomide (VRd), thalidomide (VTd) or cyclophosphamide (VCd) [10]. These medical treatments have resulted in drastic improvements in survival rates, life expectancy and quality of life for multiple myeloma patients. Overall response rates are over 80% with progression-free survival of around 50 months [11-12]. Furthermore, the integration of anti-osteoclastogenic therapies, such as bisphosphonates (zoledronic acid), and RANK-ligand inhibitors (denosumab) has approximately halved the risk of fractures [13].

The pathogenesis of multiple myeloma results from an imbalance of osteoblastic and osteoclastic bone activity with the latter dominating [14]. Consequently, over 80% of patients with multiple myeloma will suffer from destructive lesions of the bone at the point of diagnosis; These tend to be lytic in nature and <20mm in diameter [15]. Lesions occur in “the vertebrae (66% of patients), ribs (45%), skull (40%) and pelvis (30%)” [16] and require whole-body MRI, positron emission tomography-computed tomography (PET-CT) or low-dose whole-body CT ideally to diagnose. Recent studies have found MRI to have similar bony lesion detection rates when compared with CT/PET in all aforementioned areas except the ribs and skull where additional X-rays are recommended [17]. MRI is further indicated to rule out spinal cord compression and guide surgical intervention in those with spinal lesions [18].

The lytic lesions result in painful bones (73%) and impending pathological fractures (>50%) during the course of the disease [19]. This bone pain is usually managed with analgesia, chemotherapy and bisphosphonates; however, the risk of pathological fracture remains high. For those impending pathological fractures of the long bones, surgical intervention via intramedullary nailing provides a solution associated with reduced blood loss and morbidity [20]. Furthermore, prophylactic nailing was found to result in increased survival and ambulation rates six months post-surgery [21]. In 1989, Hilton Mirels proposed a system to "quantify the risk of sustaining a pathological fracture through a metastatic lesion in a long bone". This system was based on the site, nature, size and pain associated with the lesion, as seen in Table 1. High-risk lesions (scoring ≥9/12 on Mirels' scale) indicated prophylactic nailing prior to irradiation as seen in Table 2 [22]. Mirels’ system was found to be “reproducible, valid and more sensitive than clinical judgement across all experience levels” [23]. A 2003 report, however, found a lack of objectivity and reproducibility in the system [24]. Additionally, it was devised during an era in which myeloma therapies were limited. Furthermore, there are complications associated with nailing including wound dehiscence and infections, which further prolong recovery [25]. For these reasons, there has recently been a shift from managing myeloma patients, especially elderly patients experiencing their first relapse, with osteolytic lesions via instrumented surgical intervention to the aforementioned combination of steroids and chemotherapy as well as the use of minimally invasive surgery such as kyphoplasty and bracing to manage pain [26]. Thus, it is vital to take a patient-by-patient approach when considering whether medical therapy is preferable to prophylactic surgical intervention in high-risk lesions.

**Table 1:** Mirels' scoring system for lytic lesions of long bones Adapted from [27]

Score	Site	Nature	Size	Pain
1	Upper extremity	Blastic	<1/3	Mild
2	Lower extremity	Mixed lytic and blastic	1/3 – 2/3	Moderate
3	Peritrochanteric	Lytic	>2/3	Functional

**Table 2:** Clinical recommendations for the management of lytic lesions of long bones based upon Mirels' scoring system Adapted from [27]

Risk of Pathological Fracture	Mirels’ scale point total	Mirels’ treatment recommendations
Impending	≥9	Prophylactic stabilisation
Borderline	8	Consider stabilisation
Not impending	≤7	Non-operative care

## Conclusions

Multiple myeloma often warrants surgery in order to treat impending pathological fractures of long bones such as the femur; This is more so the case in those lesions graded ≥9/12 on Mirels’ score. Our case, however, shows that lytic lesions, even those indicating prophylactic nailing as per Mirels’ score, may be managed and treated effectively through the use of targeted chemotherapy and bisphosphonates thus obviating intramedullary nailing and the complications and risks associated with this. Furthermore, our case is in keeping with current literature describing the limitations of Mirels’ classification system and the potential need for an updated system. Further research should be conducted into the safety and efficacy of a purely medical treatment regimen in patients with high Mirels’ score lesions, similar to our cognizant decrease in instrumented spinal surgery. If this research shows significant benefits to purely medical management over surgical management, consideration could be taken as to whether prophylactic femoral nailing is necessary despite indications.

## References

[1] Rasch S, Lund T, Asmussen JT, Lerberg Nielsen A, Faebo Larsen R, Østerheden Andersen M, Abildgaard N (2020). Multiple myeloma associated bone disease. Cancers (Basel).

[2] Hameed A, Brady JJ, Dowling P, Clynes M, O'Gorman P (2014). Bone disease in multiple myeloma: pathophysiology and management. Cancer Growth Metastasis.

[3] Agarwal MG, Nayak P (2015). Management of skeletal metastases: an orthopaedic surgeon's guide. Indian J Orthop.

[4] Molloy S, Lai M, Pratt G (2015). Optimizing the management of patients with spinal myeloma disease. Br J Haematol.

[5] Greipp PR, San Miguel J, Durie BG (2005). International staging system for multiple myeloma. J Clin Oncol.

[6] Durie BG, Harousseau JL, Miguel JS (2006). International uniform response criteria for multiple myeloma. Leukemia.

[7] Palumbo A, Anderson K (2011). Multiple myeloma. N Engl J Med.

[8] Mateos MV, Landgren O (2016). MGUS and smoldering multiple myeloma: diagnosis and epidemiology. Cancer Treat Res.

[9] Rajkumar SV, Kumar S (2020). Multiple myeloma current treatment algorithms. Blood Cancer J.

[10] Rajkumar SV (2019). Multiple myeloma: every year a new standard?. Hematol Oncol.

[11] Rosiñol L, Oriol A, Teruel AI (2012). Superiority of bortezomib, thalidomide, and dexamethasone (VTD) as induction pretransplantation therapy in multiple myeloma: a randomized phase 3 PETHEMA/GEM study. Blood.

[12] Cavo M, Tacchetti P, Patriarca F (2008). Superior complete response rate and progression-free survival after autologous transplantation with up-front velcade-thalidomide- dexamethasone compared with thalidomide-dexamethasone in newly diagnosed multiple myeloma. Blood.

[13] Terpos E, Sezer O, Croucher PI (2009). The use of bisphosphonates in multiple myeloma: recommendations of an expert panel on behalf of the European Myeloma Network. Ann Oncol.

[14] Yeh HS, Berenson JR (2006). Treatment for myeloma bone disease. Clin Cancer Res.

[15] Lütje S, de Rooy JW, Croockewit S, Koedam E, Oyen WJ, Raymakers RA (2009). Role of radiography, MRI and FDG-PET/CT in diagnosing, staging and therapeutical evaluation of patients with multiple myeloma. Ann Hematol.

[16] Collins CD (2004). Multiple myeloma. Cancer Imaging.

[17] Regelink JC, Minnema MC, Terpos E (2013). Comparison of modern and conventional imaging techniques in establishing multiple myeloma-related bone disease: a systematic review. Br J Haematol.

[18] Rajkumar SV, Kumar S (2016). Multiple myeloma: diagnosis and treatment. Mayo Clin Proc.

[19] The Surgeon's Committee of the Chinese Myeloma Working Group of the International Myeloma Foundation (2016). Consensus on surgical management of myeloma bone disease. Orthop Surg.

[20] Obert L, Jarry A, Lepage D, Jeunet L, Tropet Y, Vichard P, Garbuio P (2005). Centromedullary nailing of the femur for bone metastasis: clinical and radiological evaluation using the Tokuhashi score in 24 patients [Article in French]. Rev Chir Orthop Reparatrice Appar Mot.

[21] Arvinius C, Parra JL, Mateo LS, Maroto RG, Borrego AF, Stern LL (2014). Benefits of early intramedullary nailing in femoral metastases. Int Orthop.

[22] Mirels H (2003). Metastatic disease in long bones: a proposed scoring system for diagnosing impending pathologic fractures. 1989. Clin Orthop Relat Res.

[23] Damron TA, Morgan H, Prakash D, Grant W, Aronowitz J, Heiner J (2003). Critical evaluation of Mirels' rating system for impending pathologic fractures. Clin Orthop Relat Res.

[24] Howard EL, Shepherd KL, Cribb G, Cool P (2018). The validity of the Mirels score for predicting impending pathological fractures of the lower limb. Bone Joint J.

[25] Angelini A, Trovarelli G, Berizzi A, Pala E, Breda A, Maraldi M, Ruggieri P (2018). Treatment of pathologic fractures of the proximal femur. Injury.

[26] van de Donk NWCJ, Pawlyn C, Yong KL (2021). Multiple myeloma. Lancet.

[27] Jawad MU, Scully SP (2010). In brief: classifications in brief: Mirels' classification: metastatic disease in long bones and impending pathologic fracture. Clin Orthop Relat Res.

